# Asking What Matters Is What Matters to Hospitalized Older Adults

**DOI:** 10.1093/geroni/igaa057.1938

**Published:** 2020-12-16

**Authors:** Vikki Rompala, Erin Emery-Tiburcio, Carline Guerrier

**Affiliations:** Rush University Medical Center, Chicago, Illinois, United States

## Abstract

The 4Ms of an Age-Friendly Health System place What Matters at the center of optimal care for older adults. Nurses at Rush have asked every medical inpatient What Matters early in their hospital stay since May, 2018. Responses were recorded in tablet software and on patient room white boards. What Matters responses recorded electronically were stratified by age and ethnicity. Qualitative data analysis of responses (n=660) was conducted using In-Vivo software by three raters. Themes in responses include: going home; comfort, including pain control and breathing more easily; effective staff/patient communication; compassionate care; and mobility. Patient satisfaction data for the first year showed an average 2.6% increase in satisfaction in nurses listening to the patient, and average 3.6% increase in satisfaction in nurses explaining things in an understandable way. Both increases were statistically significant. Implications of this practice for health systems improving age-friendly care will be discussed.

